# Identification and management of adults with asthma prone to exacerbations: can we do better?

**DOI:** 10.1186/1471-2466-8-27

**Published:** 2008-12-30

**Authors:** Neil C Thomson, Rekha Chaudhuri

**Affiliations:** 1 Respiratory Medicine Section, Division of Immunology, Infection and Inflammation, University of Glasgow, Glasgow, UK

## Abstract

Exacerbations are a major cause of morbidity in asthma and generate high health costs. Identification and management of adults with asthma who are prone to exacerbations is of considerable importance as by this means it should be possible to reduce the number of patients who currently experience inadequately controlled disease. Exacerbations occur most frequently in individuals with severe disease. Other risk factors include a history of a recent exacerbation, co-morbidities such as a raised body mass index and psychological problems as well as current smoking and lower socio-economic status. A low FEV_1_, particularly if combined with the additional information from questionnaires helps predict exacerbations. Despite the association between these risk factors and exacerbations it remains difficult to accurately predict in an individual patient with asthma whether they will go on to develop an exacerbation in the future. A major aim of international guidelines on the management of asthma is to prevent future risks of exacerbations, but some patients, particularly those with severe disease, respond poorly to current therapies and continue to experience recurrent exacerbations.

There is an unmet need for improved management strategies and drugs targeted at preventing asthma exacerbations. Monitoring induced sputum eosinophil cell counts is helpful in preventing exacerbations in some patient with severe asthma. Future developments are likely to include the identification of better biomarkers to predict exacerbations or the cause of exacerbations, augmentation of the immunological response to viruses at the time of the exacerbation, the use of telemonitoring in patients with severe asthma and the development of improved therapies targeted at reducing exacerbations.

## Background

A major goal of international guidelines on the management of asthma [1] is to achieve control of current symptoms, lung function and reliever inhaler use and to prevent future risks of exacerbations and decline in lung function [2]. Despite the widespread dissemination of asthma guidelines many patients have inadequately controlled disease [3] and experience frequent exacerbations of asthma [4,5]. Exacerbations are associated with an accelerated decline in lung function [6], generate high health costs [7] and are the main cause of mortality in asthma. The identification and appropriate management of adults with asthma who are prone to exacerbations is of considerable importance as by this means it should be possible to reduce the large number of patients who currently experience uncontrolled disease.

## Definition and risk factors for exacerbations

Severe exacerbations, defined as the need for courses of high dose corticosteroids or hospitalization because of asthma, occur most commonly in patients with severe asthma. This group can experience exacerbation rates ranging from 1.5 [8] to over 4 exacerbations per year [4]. Information on previous asthma control, co-morbidities and demographic factors as well as physiological and inflammatory biomarkers may help identify some individuals prone to exacerbations. These factors are often associated with severe asthma. A history of a recent exacerbation within the last 3 months is associated with a considerably increased risk of a future exacerbation [relative risk (RR) 3.7] [9]. Several co-morbidities in patients with difficult-to-treat asthma are associated with recurrent exacerbations including severe nasal sinus disease [adjusted odds ratio (OR) 3.7], gastro-esophageal reflux (OR 4.9), recurrent respiratory infections (OR 6.9), psychological problems (OR 10.8) and obstructive sleep apnea (OR 3.4) [10]. Current smokers with asthma are more likely to experience exacerbations compared to non-smokers with asthma [11]. A raised body mass index is also a risk factor for exacerbations [RR 1.7 (1.2–2.3)] [9] and hospitalization because of asthma [12]. Hospital admission rates for asthma in the US are associated with lower socio-economic status and are higher in black and Hispanic patients with asthma compared to whites [13]. A low pre-bronchodilator FEV_1 _of 60 to 80% of predicted gives a 2.4-fold increased risk of an exacerbation, which rises to 4.6-fold increased risk when the pre-bronchodilator FEV_1 _is < 60% of predicted [14]. Addition of information gained from questionnaires, including a history of pet ownership, increases the likelihood of identifying exacerbations over the next 30 months to a medium risk (RR 3.0) or high risk (RR 11) [14].

The categorisation of patients with severe asthma by cluster analyses into different phenotypes termed early onset atopic asthma, obese non-eosinophilic asthma, early symptoms predominant asthma and inflammation predominant asthma found that exacerbation rates were high in each of the distinct categories, but that no sub-group was more prone to exacerbations [4]. Thus despite the association between known risk factors and exacerbations it remains difficult to accurately predict in an individual patient with asthma whether they will go on to develop an exacerbation in the future.

## Management of patients prone to exacerbations

Several pharmacological and non-pharmacological management approaches are likely to be effective in preventing exacerbations. Current drugs therapies for asthma, particularly inhaled corticosteroids alone or in combination with long-acting beta_2 _agonists, but also leukotriene modifiers and omalizumab all reduce the rate of asthma exacerbations [1]. Different ways of using currently available medication may reduce exacerbation rates. The SMART approach, which involves using both budesonide and formoterol given as needed, reduces the frequency of severe exacerbations in patients receiving regular combination therapy [15], although the value of this approach in patients with severe asthma is less clear. The heterogeneity of the therapeutic response to corticosteroids and to other drug therapies for asthma means that some patients respond poorly to current treatments and continue to experience recurrent exacerbations [5], probably due to a combination of genetic and environmental factors [16] as well as poor adherence to drug therapy. Written individualized management plans, when combined with regular review, improve asthma control including reduced hospitalization and attendance at emergency rooms for exacerbations [17].

The effect of treating co-morbidities associated with severe asthma, targeting smokers with asthma to quit smoking [18] or patients with a high BMI to lose weight [19], may result in improvements in indices of current asthma control. Future studies are needed to assess whether these interventions reduce exacerbation rates. Avoidance of trigger factors such as allergens, non-steroidal anti-inflammatory agents or occupational agents in sensitive individuals, as well as exposure to environmental irritants such as passive smoke, is likely to prevent some exacerbations [1].

## Can we do better?

Taken together, there is considerable evidence to indicate a need for improved methods both to identify adults with asthma who are prone to exacerbations and also to identify the early development and cause of an exacerbation. There is also a need for better management strategies and drugs targeted at treating and preventing exacerbations.

Monitoring biomarkers of airway inflammation may have a role in reducing exacerbation rates in selected patients. Treatment based on serial sputum eosinophil count measurements, prevents exacerbations in patients with severe asthma [20,21]. Serial exhaled nitric oxide measurements however, does not decrease exacerbation rates in 12–20 year olds [22] or in adults followed up for one year [23].

In future, it may be possible to use genetic markers to identify exacerbators. For example, in children and young adults with asthma the risk of asthma exacerbations is associated with filagrin null mutations [24] and IL-10 polymorphisms [25]. The complex and fluctuating interaction between environmental, immunological and mechanical factors on the risk of future exacerbations may require the use of sophisticated analytical methods to assess risk [26]. The majority of asthma exacerbations are caused by respiratory rhinovirus infections [27]. Recent studies have demonstrated that patients with asthma are more susceptible to the clinical and inflammatory adverse effects of respiratory viruses due to augmented Th2 or impaired Th1 or IL-10 immunity [28]. These findings suggest that one approach in the future to the management of exacerbation may be through immunological augmentation with interferons at the time of the exacerbation [27] or by the use of specific anti-viral therapies. Telemonitoring of patients with severe asthma may be an advance that could identify worsening asthma control at an earlier stage, but evidence for this is still awaited. In the future, biological therapies, such as anti-IL13, current drugs, such as macrolides, or novel treatments, such as bronchial thermoplasty [29] may prove useful approaches in reducing exacerbations in some patients with severe asthma. Hopefully future research in asthma exacerbations will translate into improved levels of asthma control within the population.

## Pre-publication history

The pre-publication history for this paper can be accessed here:


